# Spatially encoded fast single-molecule fluorescence spectroscopy with full field-of-view

**DOI:** 10.1038/s41598-017-10837-6

**Published:** 2017-09-08

**Authors:** Jialei Tang, Yangyang Sun, Shuo Pang, Kyu Young Han

**Affiliations:** CREOL, The College of Optics and Photonics, University of Central Florida, Orlando, Florida USA

## Abstract

We report a simple single-molecule fluorescence imaging method that increases the temporal resolution of any type of array detector by >5-fold with full field-of-view. We spread single-molecule spots to adjacent pixels by rotating a mirror in the detection path during the exposure time of a single frame, which encodes temporal information into the spatial domain. Our approach allowed us to monitor fast blinking of an organic dye, the dissociation kinetics of very short DNA and conformational changes of biomolecules with much improved temporal resolution than the conventional method. Our technique is useful when a large field-of-view is required, for example, in the case of weakly interacting biomolecules or cellular imaging.

## Introduction

A camera-based detector has been widely used to study many biological systems with single-molecule fluorescence techniques because of its high sensitivity and high-throughput^1, 2^. However, in spite of remarkable technical advancements over the last decade, the maximum frame rate of the detector, i.e. electron multiplying charge-coupled device (EMCCD) or scientific complementary metal-oxide semiconductor (sCMOS) camera is still at 50–100 frames per second. Cropping the field-of-view (FOV) is the simplest way to improve temporal resolution, however, it decreases the number of molecules that can be observed simultaneously^3^. This is especially disadvantageous for studying weakly interacting biomolecules because the complex formation will occur very rarely^4^. For example, assuming that the binding rate is 10^6^ M^−1^ s^−1^ and the accessible concentration of fluorophore-labeled biomolecules is 10 nM in TIRF microscopy, the rate of the complex formation amounts to ~10^−2^ s^−1^. Alternatively, stroboscopic illumination could be used, but it loses the dynamic information during which the excitation pulses are turned off^5^. Thus, a technique that uses a full chip but provides higher temporal resolution than a given frame rate is still lacking.

**Figure 1:**
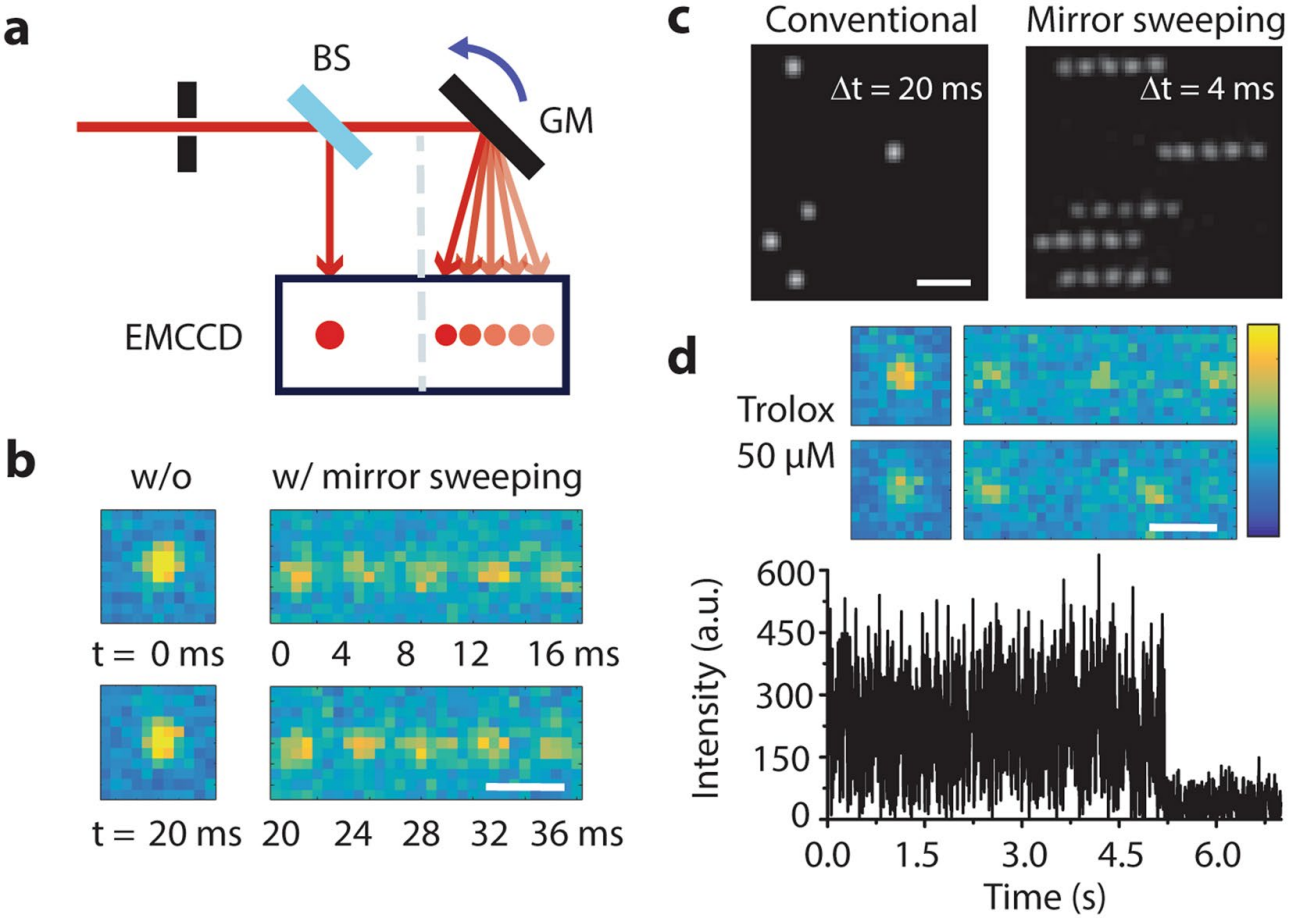
Spatially encoded fast single molecule spectroscopy with full field-of-view. (**a**) Schematic of spatially encoding temporal information in single-molecule imaging. The galvo mirror was rotated during the camera exposure time. BS, beam splitter; GM, galvo mirror. (**b**,**c**) Single-molecule images of Atto647N attached to single-stranded DNA obtained by conventional (left) and high temporal resolution imaging (right) at 2 mM Trolox. Average of ten frames in (**c**). (**d**) Blinking dynamics of Atto647N at 50 µM Trolox. Single-molecule raw images (top) and fluorescence time traces (bottom) at 4-ms time resolution. Scale bars, 1 µm (**b,d**) and 2 µm (**c**).

Interestingly, high-speed photography overcame frame rate problem long time ago by means of a rapidly rotating mirror that spreads images to multiple cameras at different time points^6^. Inspired by that technique, we devised a simple method that encodes temporal information into the spatial domain by exploiting the sparsity of single-molecule samples similar to spatially encoding a spectrum^7^ or an axial position of single molecules^8^. To do so, we used an objective-type total internal reflection fluorescence microscope with EMCCD, in which a beam-splitter and a single-axis galvo mirror were installed between two relay lenses (Fig. 1a, Supplementary Fig. 1). During the exposure period (*∆t* = 20 ms), we swept the galvo mirror in *N* steps (for instance, *N* = 5), which resulted in *N* equally spaced single-molecule spots produced on the image plane with higher temporal resolution (*∆t*/*N* = 4 ms) (Fig. 1b,c, Supplementary Fig. 2). The number of steps and the distance between the spots were controlled by a waveform generator (Supplementary Fig. 3). The beam splitter was used to obtain unmodified images either for comparison or for determination of an initial point of the event.

## Results

As a proof-of-principle experiment, we first recorded fluorescence transients of Atto647N labeled to a single-stranded DNA on the surface. Photoblinking happens when the fluorophore intermittently switches back and forth between a bright fluorescent state and a dark nonfluorescent state on a timescale that ranges from microseconds to minutes, depending on different dark states^9^. However, an addition of Trolox with oxygen removal effectively prevents photoblinking and photobleaching of many fluorophores^10^ due to a combinatorial effect of its reducing and oxidizing properties^11^. For Atto647N, the on-count and the off-time are dependent on the concentration of the reducer (Trolox) and oxidizer (a quinone derivative of Trolox), respectively, and thus, its blinking dynamics could be readily tuned by changing the concentration of Trolox. As shown in Fig. 1d, our method enabled us to observe clearly the blinking dynamics of the fluorophore at low concentration of Trolox, whereas the temporal resolution of 20 ms by the conventional method did not allow measuring the blinking dynamics (Supplementary Fig. 4). We used auto-correlation analysis to determine the off-time^12, 13^, which was 4.6 ± 0.8 ms and 3.5 ± 0.5 ms at 50 μM and 200 μM Trolox, respectively (Supplementary Fig. 5). Both methods exhibited steady fluorescence intensity at high concentration of Trolox (Supplementary Fig. 6). Note that the fast blinking dynamics with millisecond time scale has been observed generally through confocal microscopy with a single pixel detector^11^.

Since signal-to-noise ratio (SNR) is a crucial criterion in fast single-molecule measurements, we analyzed it using fluorescence trajectories of Atto647N at 2 mM of Trolox (*n* > 50). Here we defined SNR_B_ = (*I*_S_ − *I*_B_)/*σ*_B_ and SNR_S_ = (*I*_S_ − *I*_B_)/*σ*_s_, where *I*_S_ is the mean of signal intensity, *I*_B_ is the mean of background intensity, *σ*_s_ and *σ*_B_ are their respective standard deviations. The measured SNR_B_ and SNR_S_ were 34.0 ± 5.2 and 11.5 ± 3.1 at 20 ms resolution whereas they were 8.3 ± 1.4 and 4.1 ± 0.6 at 4 ms resolution (Supplementary Fig. 7). At given illumination intensity, the average photobleaching time was 69.7 s.

**Figure 2:**
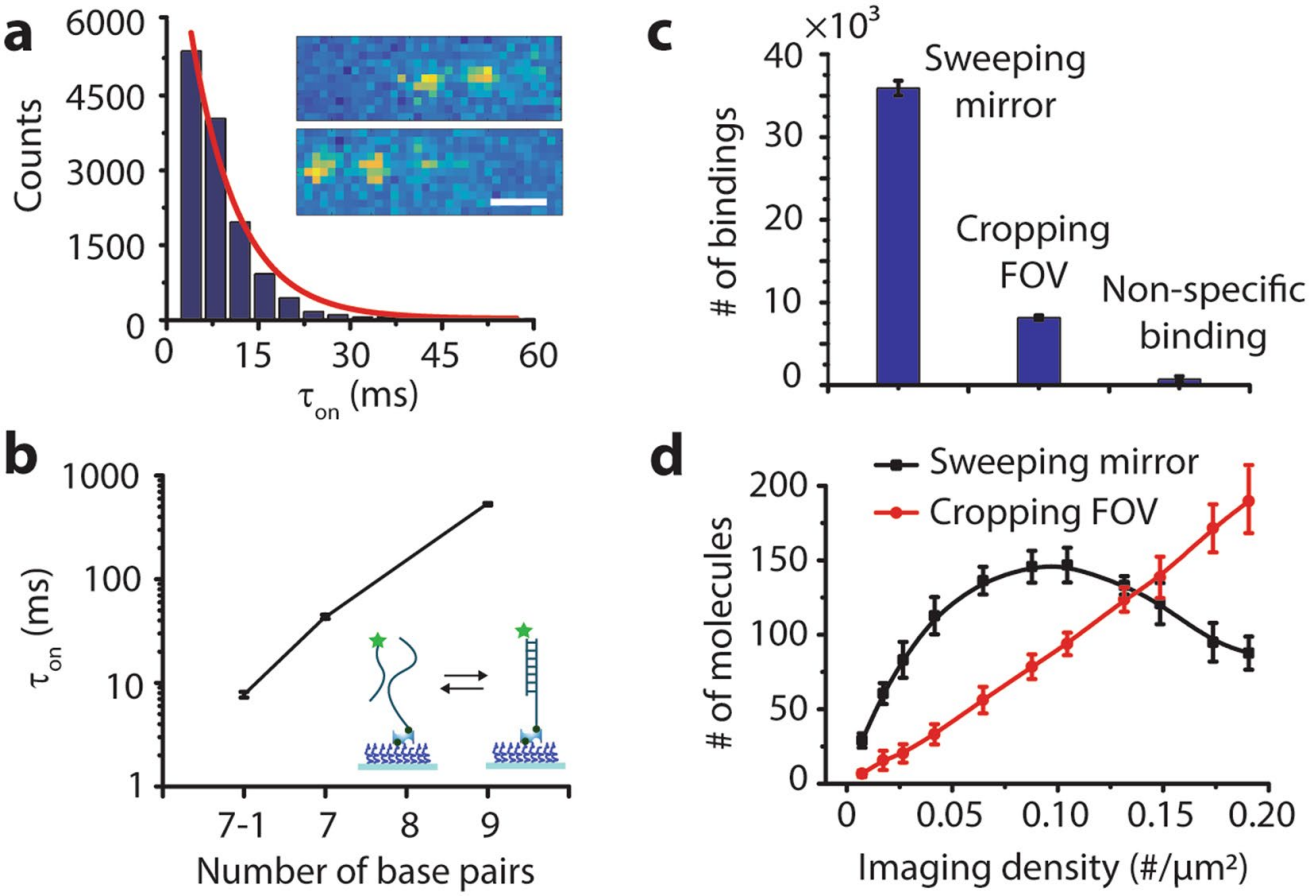
Binding and unbinding kinetics of short double-stranded DNA. (**a**) Dwell time histogram of 7-1 bp over five movies of 2,000 frames. Inset: Single-molecule raw images of binding and unbinding events of Cy3B-labeled single-stranded DNA. (**b**) Dependence of the on-time (τ_on_) on the length of DNA duplexes from five movies. The on-time of 7-1, 7 and 9 bp were 7.7 ± 0.5 ms, 43.5 ± 2 ms and 530 ± 12 ms, respectively. Data are the mean ± standard deviation from >6,000 binding events. (**c**) The number of binding events of 7-1 bp with sweeping mirror or cropping FOV from five movies. Scale bars, 1 µm. (**d**) The number of resolvable molecules at different imaging densities.

Next, we measured the dissociation kinetics of short double-stranded DNA (*n* < 8 nt) as an example of weakly interacting biomolecules. The binding (association) and unbinding (dissociation) of double-stranded DNA (dsDNA) through base-pairing is an essential process in DNA replication and repair^14^, and those processes are also used for point accumulation imaging in nanoscale topography (DNA-PAINT), leading to super-resolution fluorescence imaging for fixed samples^15^. However, the kinetics of extremely short dsDNA are still elusive due to the fact that (i) the dissociation rate is fast (*k*_off_ > 20 s^−1^)^15^, and (ii) the association rate (10^4^~10^6^ M^−1^s^−1^) limits the number of observable binding events unless the molecules are confined to the vesicle^14^ or trapped electro-kinetically^16^. A previous study reported at least seven contiguous base pairs were required for fast annealing^14^ and the unbinding rate *k*_off_ (1/τ_on_) of 9-bp DNA with a mismatched sequence was ~5 s^−1^. Here, our method allowed us to observe the rare binding events of the 7-bp DNA strand with a 1-bp mismatch at one end of the duplex (7-1 bp) labeled with Cy3B (Fig. 2a, Supplementary Fig. 8), exhibiting the on-time (τ_on_ = 1/*k*_off_) of 7.7 ± 0.5 ms. We excluded the possibility that on/off fluorescence signals were due to the photoblinking of Cy3B (Supplementary Fig. 9). Our results were in good agreement with the base pair length dependence of the on-time as previously reported (Fig. 2b)^15^. Compared to cropping FOV, our method yielded 4.5-fold higher throughput (Fig. 2c) and allowed for the detection of more molecules for imaging densities up to 0.13 molecules/μm^2^ (Fig. 2d and Supplementary Fig. 10). But at higher imaging density, overlapping between the swept spots results in diminished throughput.

**Figure 3:**
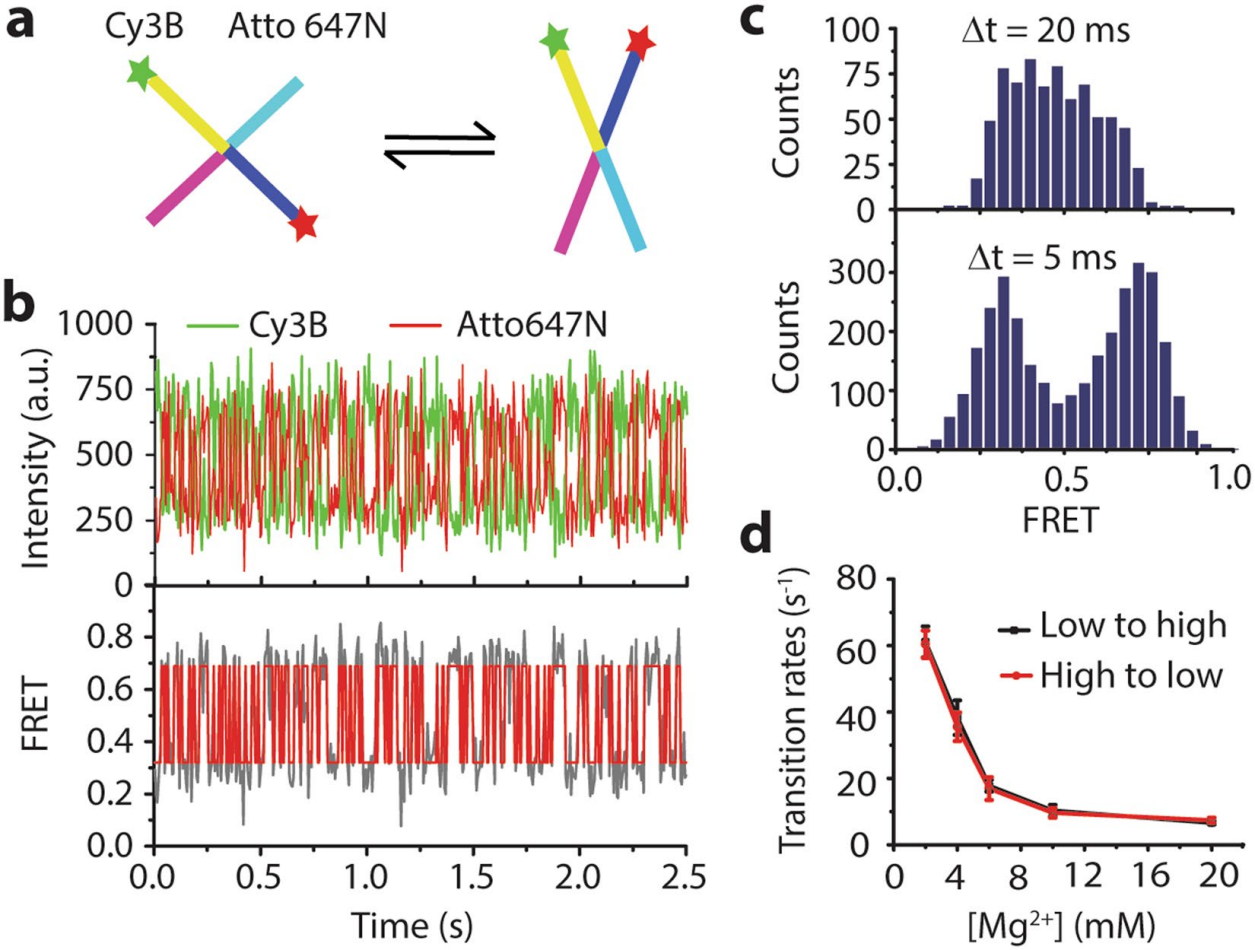
Holliday junction dynamics. (**a**) Cartoon of Holliday junction dynamics. (**b**) Single-molecule fluorescence intensity (top) and FRET time traces (bottom) of Holliday junction showing rapid conformational changes at 2 mM Mg^2+^. In FRET time trace (solid gray line), a red solid line is a fit with hidden Markov model. (**c**) Single-molecule FRET histograms of HJ dynamics with 20-ms and 5-ms time resolution from ~750 and ~3,000 data points, respectively. (**d**) Transition rates of Holliday junctions at different magnesium concentrations. More than 30 molecules from 5 movies were taken.

Lastly, we monitored the conformational dynamics of biomolecules using single-molecule FRET where we slightly modified the detection setup (Supplementary Fig. 1b). In the presence of magnesium ions, a four-way DNA structure known as Holliday junction (HJ) folds itself into two possible stacked structures (Fig. 3a)^17^. Previous studies showed that the transition rates were highly dependent on DNA sequences, concentrations of counter ions and temperature^17^. Particularly, the transition rate decreases dramatically with an increase of the concentration of magnesium. In order to observe fast dynamics of HJ, we used a low magnesium concentration during the experiments. By hidden Markov model analysis, we observed fast two-state conformational changes of single HJ molecules (Fig. 3b and Supplementary Fig. 11). Two distinct FRET populations were markedly revealed at 5-ms time resolution as contrasted with a broad FRET distribution obtained at 20-ms time resolution (Fig. 3c). The transition rate at 2 mM of magnesium ions was 61.1 ± 4.7 s^−1^ (Fig. 3d) which is in agreement of with the literature^18^.

## Discussion

Our simple method demonstrated 5-fold higher temporal resolution in single-molecule fluorescence spectroscopy, which enables the study of 100–200 molecules simultaneously. Further improvement in temporal resolution can be realized with increase of the step number *N* up to 10. In addition, our method could be used at even higher imaging densities with many overlapped spots taking advantage of that the peak positions of each spot are pre-determined by the applied voltage. Note that it is applicable to not only a larger detector such as sCMOS^19^ for improved throughput, but also to improve the temporal resolution of zero-mode waveguides^20^. Our technique will be particularly useful when cropping FOV is not feasible, for example, in the case of cellular imaging.

## Methods

### Single-molecule TIRF microscope

Single-molecule imaging was carried out on an objective-type total internal reflection fluorescence (TIRF) microscope built around Olympus IX73 (Supplementary Fig. 1a) similar to previous work^3^. Combined via a polarizing beam splitter, a green laser (Cobolt 06-DPL,532 nm), for excitation of Cy3B) and a red laser (Cobolt 06-MLD, 638 nm) with a laser clean-up filter were coupled to a single mode fiber. Collimated beams from the fiber were focused onto the back focal plane of an objective lens (UPlanSApo, 100x/1.40 oil, Olympus) by a focusing lens (f = 300 mm) and reflected by a multiband dichroic mirror (Di03-R405/488/532/635, Semrock). Fluorescence emission was collected by the same objective and filtered through a double notch filter (ZET532/638 m, Chroma). Then it was passed through the tube lens (f_TL_ = 180 mm) and two relay lenses (f_3_ = 100 mm, f_4_ = 120 mm), and detected by EMCCD (iXon Ultra 897, Andor). An adjustable slit was located at the intermediate image plane to limit the final image to half of the EMCCD. A single-axis galvo mirror (GVS211, Thorlabs) placed between the relay lenses was rotated in a step-wise manner by a function generator (DG1032Z, Rigol) and it was synchronized with an output of EMCCD (Supplementary Fig. 1c). The step response time of the galvo mirror was 0.6 ms. The time lag between the steps was negligible. For the measurements of dye blinking and short stranded DNA binding/unbinding events, a beam splitter (BSS10R, 30:70 (R:T), Thorlabs) was installed in front of the galvo mirror to separate the fluorescence beam into two channels, i.e. a conventional image with 20-ms time resolution and a spatially encoded high temporal resolution image. For the single-molecule FRET experiments, the fluorescence beam was directly swept by the galvo mirror and then split into donor and acceptor channels through a dichroic beam splitter (FF640-FDi01, Semrock) (Supplementary Fig. 1b). In both cases, the separated beams were focused onto the left and right side of the EMCCD with 512 × 512 pixels corresponding to an imaging area of 68 µm × 68 µm. The exposure time was 20 ms and the lag between images was 1.7 ms. In smFRET experiments, a short-pass filter was additionally used to suppress auto-fluorescence from the coverslip.

### Temporal calibration

We generated *N*-step function by raising the voltage from 0 to *V* in *V*/(*N* − 1) millivolt increments, where *N* is the number of spots and *V* is the voltage amplitude of the final spot (Supplementary Fig. 1c). While the generated waveform was fed into the galvo mirror, fluorescent crimson beads of 200-nm diameter (F8806, Invitrogen) sparsely immobilized on the coverslip were imaged by the TIRF microscope (Supplementary Fig. 3a). The total sweeping distance (*L*) between the first and the last spots increased linearly with the voltage applied to the galvo mirror, exhibiting the slope of 0.25 pixel/mV (Supplementary Fig. 3b). For unambiguous measurements, we set a criterion that the distance between two adjacent spots, 0.25 *V*/(*N*−1) in pixel is approximately double the full width at half maximum (FWHM) of single molecule spot. For example, since the FWHM of single Atto647N was 2.56 ± 0.55 pixels, corresponding to 340 ± 73 nm, all spots were well separated when the sweeping voltage was 96 mV (24 pixels) in 5 steps case.

### Data analysis

Similar to smFRET analysis^1, 21^, we pre-selected single-molecule spots on the left side and then identified the corresponding spots on the right side by the mapping coefficients which were calculated using the fluorescent beads with *V* = 0 mV. In blinking dynamics and short dsDNA kinetics, we summed the fluorescence intensity in 7 × 7 pixels area around the peak. For the spots generated by sweeping mirror on the right side, as sliding the area horizontally with 6 pixels, we concatenated those intensities either for building time traces or for binding event judging. The signal-to-noise ratio (SNR) of our method was assessed by using fluorescence time traces of Atto647N at 2 mM Trolox for each sweeping step. We obtained average auto-correlation curves from fluorescence time trajectories (*n* > 30) and fitted them with (*k*_off_/*k*_on_)exp[−(*k*_on_ + *k*_off_)*t*], where we assumed a two-state system made of a bright and a dark state with transition rates of *k*_on_ and *k*_off_^12, 13^. For DNA binding/unbinding experiments we used either a threshold (*I* = *I*_B_ + 3 × *σ*_B_) or hidden Markov analysis^22^ to determine the binding time; however, we mainly used the former that was simple and robust to assign the binding and unbinding events. In HJ experiments, we used the first ten frames without the mirror sweeping for mapping and selecting single-molecules, and extracted the high-temporal fluorescence intensity from the mirror swept image as mentioned above. We used vbFRET software^23^ to determine the number of states and the transition rates of the acquired time traces in HJ dynamics. To avoid any interference among the sweeping spots, it was important to select good single molecule spots that do not overlap each other within the range of sweeping distance. All time traces in Figs 1, 3 and Supplementary Figures were background-subtracted. The FRET efficiency in Fig. 3 was approximated as *I*_*A*_/(*I*_*D* +_ *I*_*A*_), where *I*_*D*_ and *I*_*A*_ are fluorescence intensities of the donor and acceptor, respectively, with background and leakage-correction.

### Preparation of flow chamber

We used polyethylene glycol (PEG)-coated flow chambers as described elsewhere^1, 3^ except the experiments of Atto647N blinking dynamics.

### DNA samples

All oligonucleotides were purchased from IDT. Amino-modified oligonucleotides were labeled with Cy3B NHS (PA63101, GE Healthcare) or Atto647N NHS (18373, Sigma) in 0.1 M borate buffer (pH 8.5) and purified twice through ethanol precipitation. Please see Supporting Table 1 for complete sequence information.

### Blinking dynamics of Atto647N

A flow chamber was passivated with 1 mg/mL of biotin-labeled bovine serum albumin (A8549, Sigma) followed by rinsing and an introduction of Neutravidin (31000, Thermo Fisher). Then single-stranded DNA labeled with Atto647N (ssDNA-A647N) was immobilized to the coverslip. We used an imaging buffer consisted of 20 mM Tris (pH 8.0), 50 mM NaCl, an enzymatic oxygen scavenger system (0.8% (w/v) dextrose, 1 mg/mL glucose oxidase (G2133, Sigma), 0.04 mg/mL catalase (2190015MU, EMD Millipore)) and Trolox (sc-200810, Santa Cruz) with various concentrations. In order to increase the Trolox-quinone concentration, Trolox was prepared at least one week before the experiment. An average power of the red laser was ~12 mW at the back focal plane of the objective lens. Single molecule movies were analyzed using our custom made MATLAB scripts as described earlier^3^.

### Binding and unbinding kinetics of short double-stranded DNA

We introduced 50 µL neutravidin (20 µg/mL) to the flow chamber coated with PEG and biotin-PEG. After washing, we incubated the capture DNA (10 nM) for 1 min followed by an injection of 50 µL Cy3B labeled ssDNA (1 nM) in the imaging buffer (1 × PBS, 600 mM NaCl, 10 mM NiCl_2_, 0.8% (w/v) dextrose, 1 mg/mL glucose oxidase, 0.04 mg/mL catalase). Here we used Ni^2+^ instead of Trolox to improve the photostability of Cy3B^24^. The excitation power of 532 nm laser was ~10 mW. The voltage applied to the galvo mirror was 112 mV in 5 steps and the total sweeping distance was approximately 28 pixels. More than ten thousand independent events were recorded to build the dwell time histogram of τ_on_, exhibiting 7.7 ± 0.5 ms, 43.5 ± 2.0 ms and 530 ± 12 ms for the 7-1, 7 and 9 bp DNA strands, respectively. The total number of binding/unbinding events of the 7–1 bp DNA was ~12,500 during 2,000 frames. The number of non-specific binding events was ~800 during the same imaging time so its contribution was negligible (Fig. 2c, Supplementary Fig. 8c). We examined the possibility that on/off fluorescence signals might be attributed to photophysics. To this end, we measured fluorescence trajectories of 9 bp dsDNA labeled with Cy3B which has τ_on_ = 530 ± 12 ms. During the ON time, the blinking events of Cy3B were rarely observed at 4 ms time resolution among ~6,600 time traces. SNR_B_ and SNR_S_ of Cy3B were 8.2 ± 1.6 and 4.0 ± 0.6.

### Holliday junction dynamics

The Holliday junction sample was prepared by mixing Seq. 1,2,3 and 4 with a molar ratio of 1:0.9:1:1 (final concentration, ~5 μM each) in 10 mM Tris-HCl (pH 8.0) and 50 mM NaCl followed by slowly cooling down from 95 °C to room temperature for ~2 h. HJ molecules were immobilized on the PEG-coated coverslip and imaged with an imaging buffer (20 mM Tris (pH 8.0), 2–20 mM MgCl_2_, 50 mM NaCl, 2 mM Trolox, 0.8% (w/v) dextrose, 1 mg/mL glucose oxidase, 0.04 mg/mL catalase). We applied 96 mV to the galvo mirror (4 steps), resulting to 5-ms time resolution (Supplementary Fig. 11b). For each concentration of magnesium, more than 30 time traces of different molecules (from more than 5 movies) were used to calculate the transition rates.

## Electronic supplementary material

Supplementary Information
