# Supplementary material for: Spatially encoded fast single-molecule fluorescence spectroscopy with full field-of-view
Source: Sci Rep. 2017 Sep 8;7:10945. doi: 10.1038/s41598-017-10837-6 (PMC5591195; doi:10.1038/s41598-017-10837-6)
Supplement: Supplementary file 1 — Supplementary Information [file 41598_2017_10837_MOESM1_ESM.pdf]

Supplementary Information

Spatially encoded fast single-molecule fluorescence spectroscopy with full field-of-view

Jialei Tang, Yangyang Sun, Shuo Pang & Kyu Young Han

CREOL, The College of Optics and Photonics, University of Central Florida, Orlando, Florida, USA.
Correspondence should be addressed to K.Y.H. (kyhan@creol.ucf.edu).

Supplementary Figures

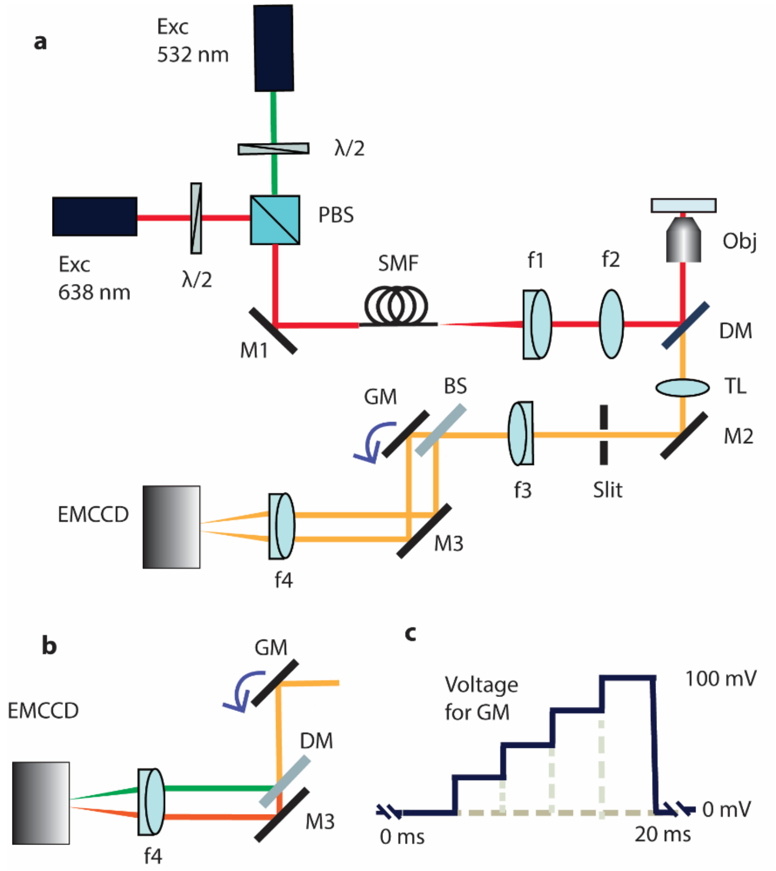

Supplementary Figure 1. Experimental scheme of spatially encoded single-molecule fluorescence spectroscopy. (a) Detailed setup of objective-type total internal reflection fluorescence (TIRF) microscope with a sweeping mirror. BS, 30:70 beam splitter; DM, dichroic mirror; f1-4, lenses; GM, galvo mirror; λ/2, half-wave plate; M1-3, mirrors; PBS, polarizing beam splitter; SMF, single mode fiber; TL, tube lens. (b) Detection path for smFRET experiment. (c) Step function of voltage for rotating the galvo mirror during the exposure of the camera.

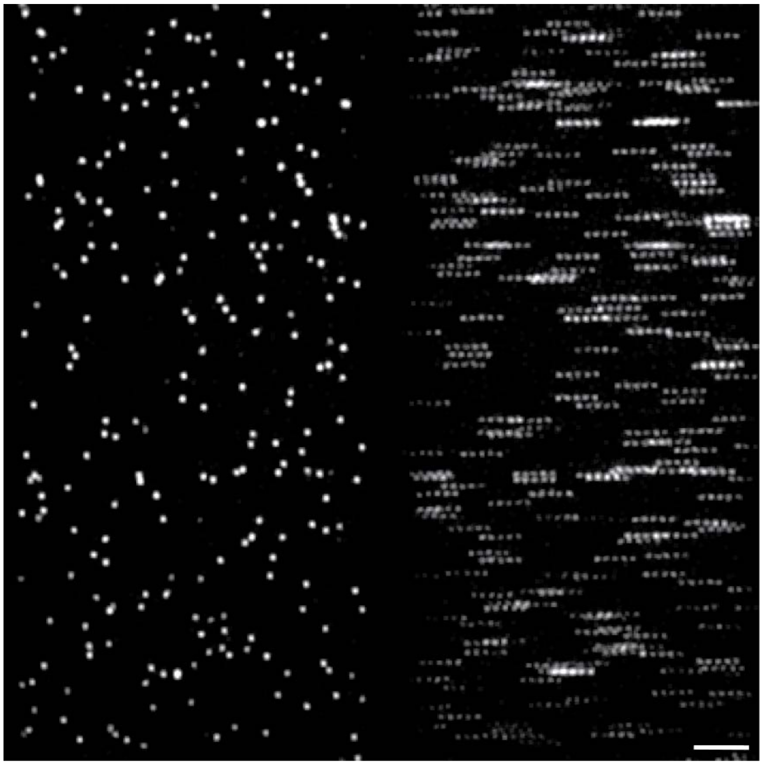

Supplementary Figure 2. Single molecule imaging of Atto647N without (left) and with sweeping mirror at 2 mM Trolox. Scale bar 5 µm.

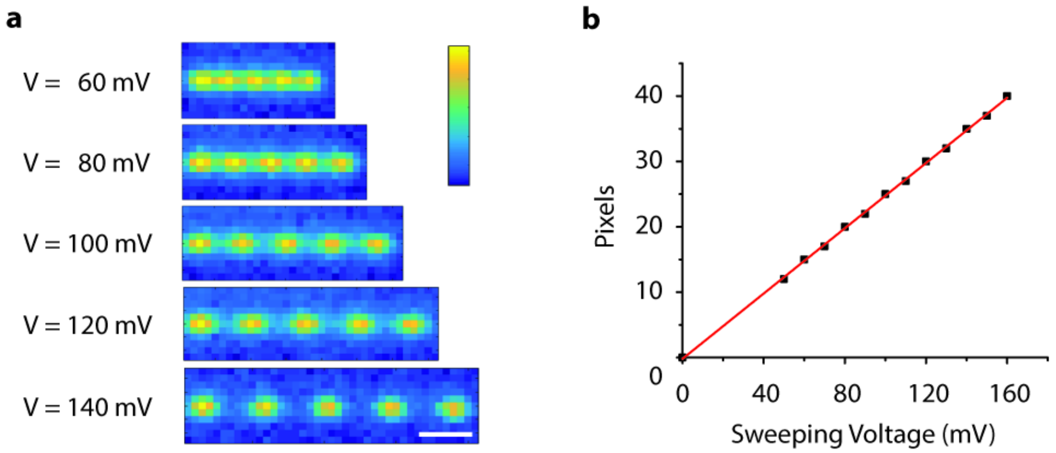

Supplementary Figure 3. Sweeping voltage dependence and calibration curve. (a) Mirror swept images of 200 nm fluorescent beads at different voltages applied to the galvo mirror. Scale bar, 1 µm. (b) Total sweeping distance in pixels as a function of the applied voltage. The red line is a linear fit for the experimental data with a slope of 0.25.

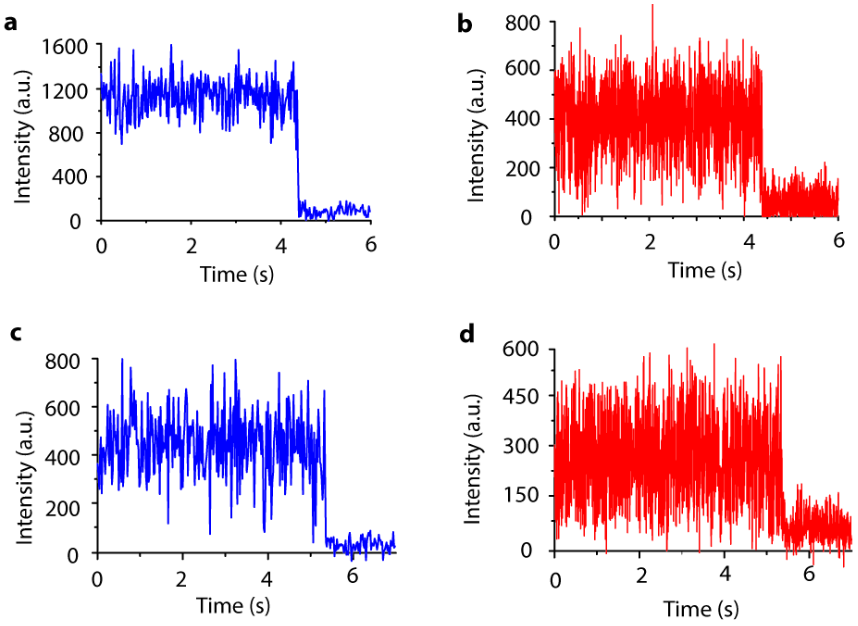

Supplementary Figure 4. Single-molecule time traces of Atto647N at different concentrations of Trolox. Time traces at 200 µM Trolox with temporal resolution of 20 ms (a) and 2.5 ms (b). Time traces at 50 µM Trolox with temporal resolution of 20 ms (c) and 4 ms (d).

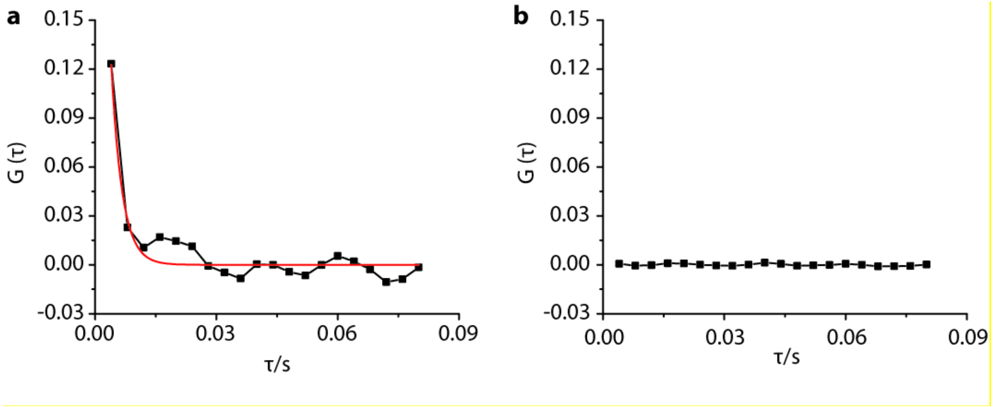

Supplementary Figure 5. Autocorrelation analysis of Atto647N blinking dynamics at 50 µM (a) and 2 mM (b) of Trolox. The red curve is the exponential fit with τ_{off} = 4.6 ± 0.8 ms.

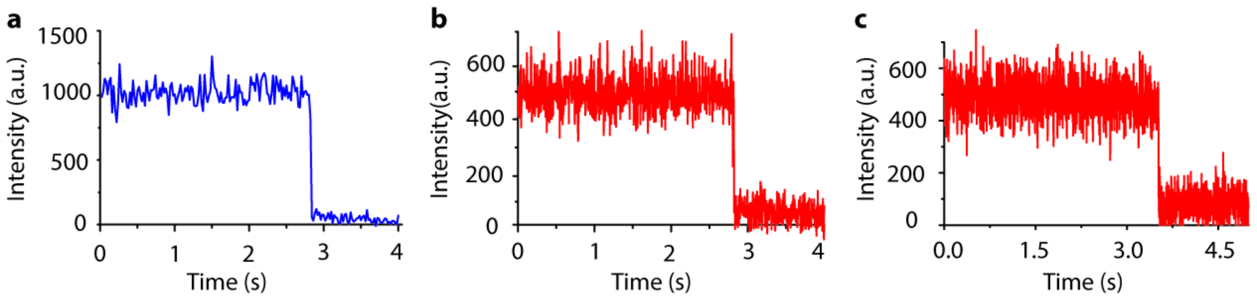

Supplementary Figure 6. Single-molecule time traces of Atto647N at 2 mM of Trolox. Time traces with temporal resolution of 20 ms (a), 4 ms (b) and 2.5 ms (c).

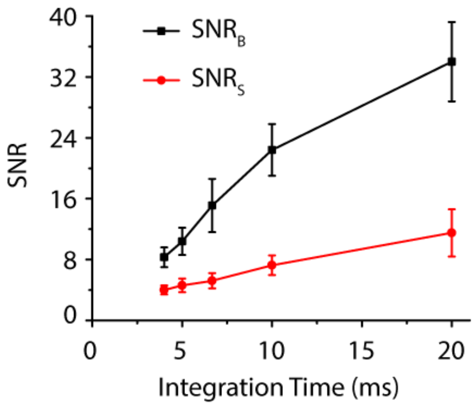

Supplementary Figure 7. Signal-to-noise ratio (SNR) of our sweeping system at different integration time. Fluorescence trajectories of Atto647N were used at 2 mM of Trolox (*n* > 50).

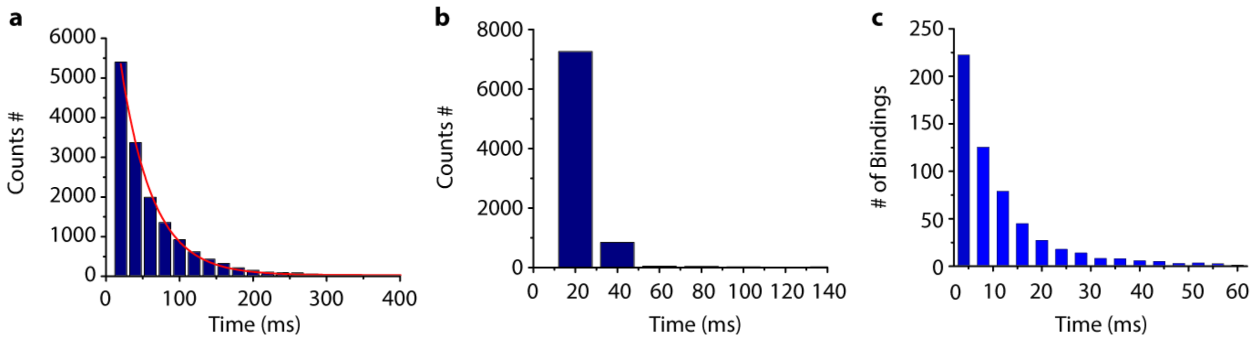

Supplementary Figure 8. Dwell time histogram of 7 bp (a) and 7-1 bp (b) DNA strands with 20-ms temporal resolution. The red curve is an exponential fit, τ_{on} = 43.5 ± 2.0 ms. (c) histogram of the non-specific binding events with 4 ms temporal resolution.

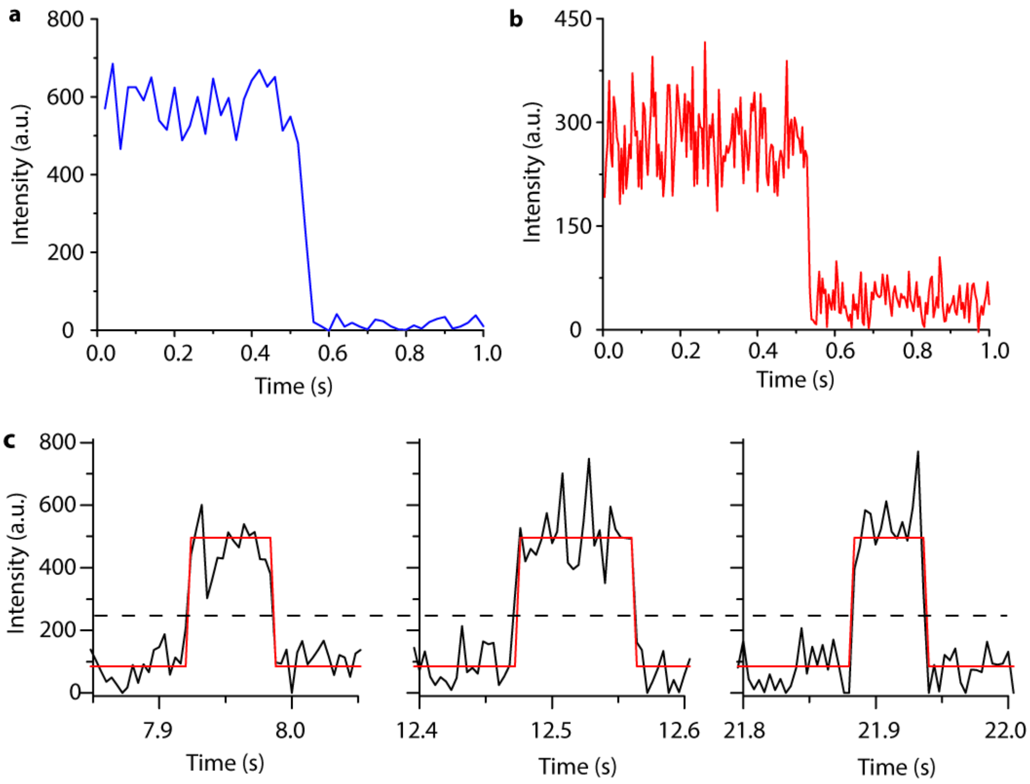

Supplementary Figure 9. Single-molecule time traces of Cy3B labeled 9 bp and 7 bp dsDNA. Time traces with temporal resolution of 20 ms (a), 4 ms (b). (c) A representative trajectory of 7 bp dsDNA binding/unbinding events with 4 ms temporal resolution. The red curves are the results of HMM analysis and the dashed line is the threshold used for judging on/off state.

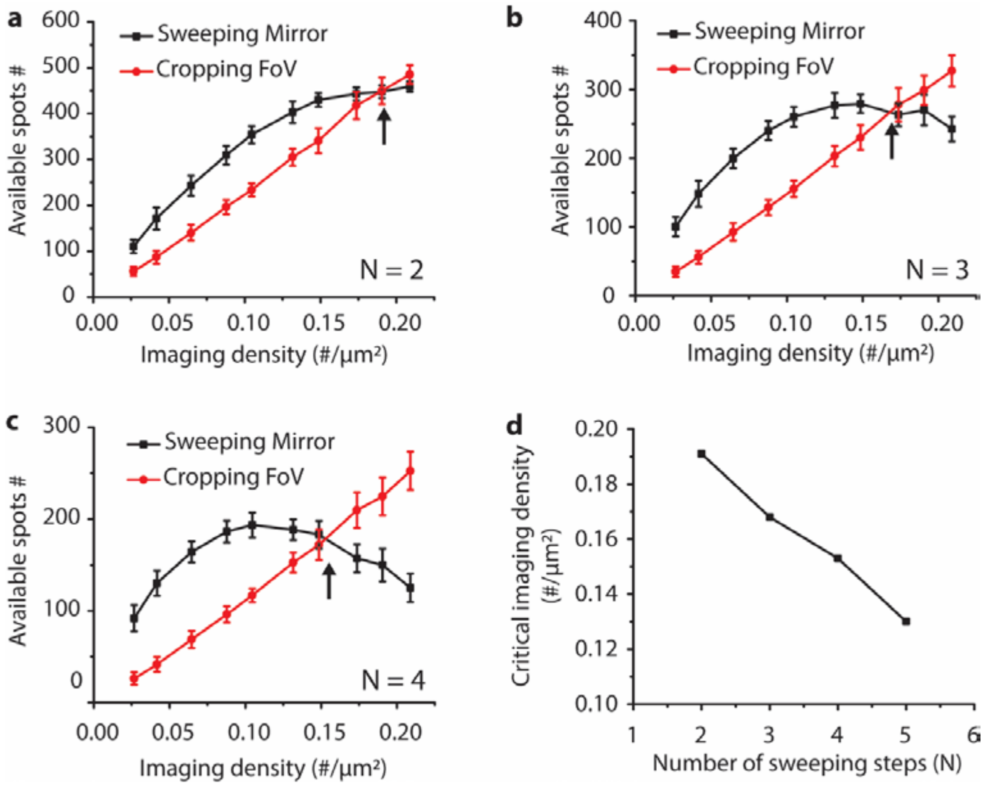

Supplementary Figure 10. Trade-off between the imaging density and the number of observable single molecules at different number of sweeping steps. Arrow marks indicate a critical imaging density where the sweeping mirror and cropping FOV show the same throughput. Atto647N-ssDNA with different concentrations were used for the analysis.

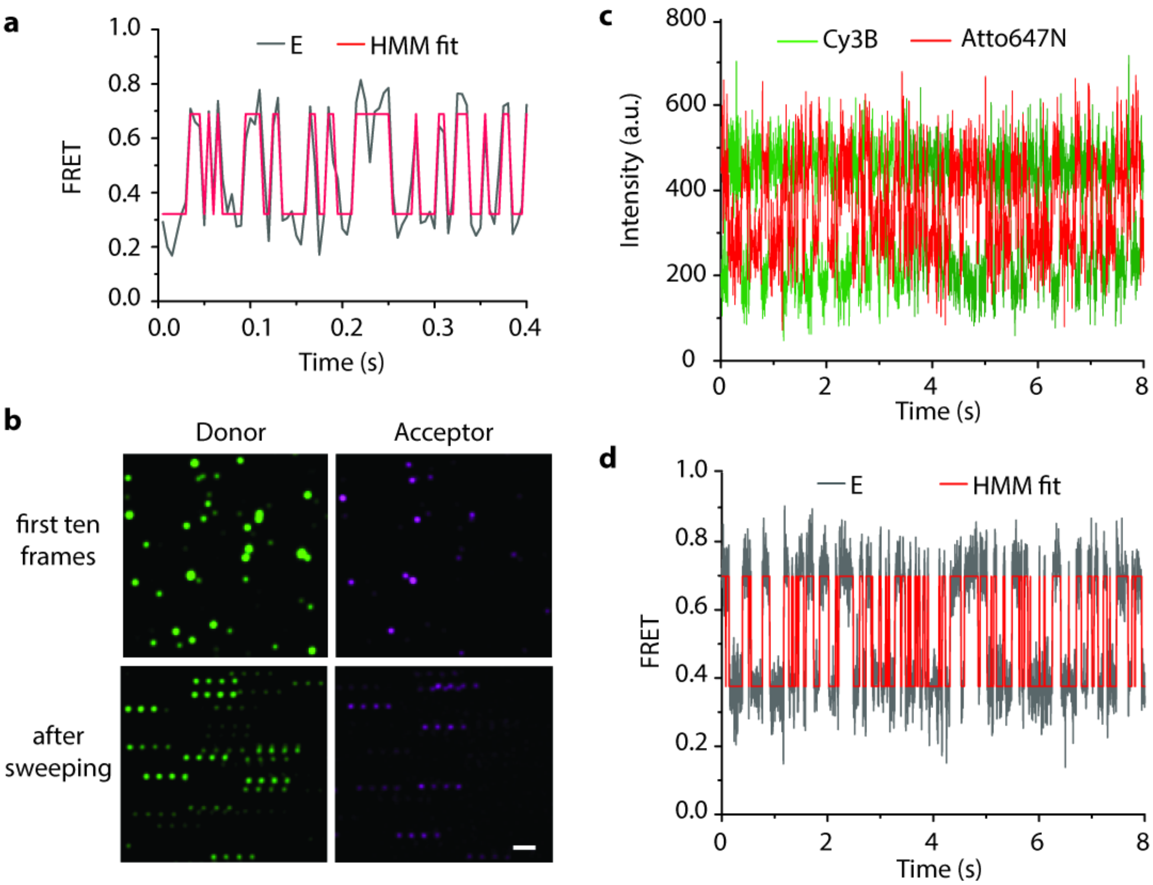

Supplementary Figure 11. Holliday junction dynamics probed by smFRET. (a) Detailed FRET time trace (green) and HMM fit (red) in Fig. 3b. (b) Experimental procedures consisted of first imaging ten frames without sweeping mirror (upper) and soon after imaging 500 frames with sweeping mirror (lower). Scale bar, 2 µm. Fluorescence intensity (c) and FRET time traces (d) of Holliday junction with 10 mM Mg^{2+} at 5 ms integration time.

Supplementary Table 1. DNA sequences.

Name	Sequence	Experiment
ssDNA_A647N	5′-/5Biosg/TGGCGACGGCAGCGAGGC/3ATTO647NN/-3′	Blinking
Capture DNA	5′-ATACATCTAGCTTTTTTTTTT/3Bio/-3	Short dsDNA binding
9 bp	5′-TAGATGTAT/Cy3B/-3′	Short dsDNA binding
7 bp	5′- GATGATT/Cy3B/-3′	Short dsDNA binding
7-1 bp	5′- **A**ATGATT/Cy3B/-3′	Short dsDNA binding
Seq1	5′-/Cy3B/CCTCCCTAGCAAGCCGCTGCTACGG-3′	HJ
Seq2	5′-CCGTAGCAGCGCGAGCGGTGGG-3′	HJ
Seq3	5′-/biotin/CCCACCGCTCGGCTCAACTGGG-3′	HJ
Seq4	5′-/Atto647N/CCCAGTTGAGCGCTTGCTAGGG-3′	HJ
